# Nanostructured Ni-Zeolite Y and Carbon Nanohorns Electrode for Sensitive Electrochemical Determination of B-Group Vitamins

**DOI:** 10.3390/ijms262110469

**Published:** 2025-10-28

**Authors:** Katarzyna Fendrych, Justyna Nyrka, Joanna Smajdor, Robert Piech, Bogusław Baś

**Affiliations:** Department of Analytical Chemistry and Biochemistry, Faculty of Materials Science and Ceramics, AGH University of Krakow, al. A. Mickiewicza 30, 30-059 Kraków, Poland; fendrych@agh.edu.pl (K.F.); smajdorj@agh.edu.pl (J.S.); bas@agh.edu.pl (B.B.)

**Keywords:** carbon nanohorns, dietary supplements, vitamin B_2_, voltammetry, zeolite-modified electrode, zeolite Y

## Abstract

This work presents the fabrication and analytical application of nanostructured Ni-zeolite (NiZY) and carbon nanohorns (CNHs) modified glassy carbon electrode (NiZY/CNHs-GCE) in the differential pulse voltammetric (DPV) determination of vitamin B_2_ (VB_2_) molecules. The synergistic combination of NiZY and CNHs significantly enhances the electrochemical performance of the sensor, as confirmed by structural, textural, morphological, and electrochemical studies. The redox behavior of VB_2_ on NiZY/CNHs-GCE was found to be adsorption-controlled, involving a two-electron, two-proton reversible reduction process. Under optimized conditions, the DPV response of NiZY/CNHs-GCEs in McIlvaine buffer solution (pH 3.4) exhibited a linearity in the VB_2_ concentration range of 0.01 to 0.20 mg L^−1^ (r = 0.9993) with a detection limit of 3.2 µg L^−1^ (8.6 × 10^−9^ mol L^−1^). Furthermore, well-resolved reduction peaks of vitamins B_2_ and B_9_ (VB_9_) enabled their simultaneous and selective detection, with linear ranges of 0.01 to 0.20 mg L^−1^ for VB_2_ and 0.01 to 0.16 mg L^−1^ for VB_9_. The proposed analytical method, characterized by high selectivity and robustness, was successfully applied in the determination of both vitamins in commercially available dietary supplements, achieving relative errors within −6.2% to 2.7%.

## 1. Introduction

The vitamin B_2_ molecule (VB_2_), commonly known as riboflavin, is an essential water-soluble micronutrient that plays a crucial role in cellular metabolism, acting as a precursor of the coenzymes flavin mononucleotide (FMN) and flavin adenine dinucleotide (FAD). These cofactors participate in various biochemical processes, including cell respiration, antioxidant defense, and the metabolism of fats, proteins, and carbohydrates [1,2]. Since the human organism cannot synthesize riboflavin, it must be obtained from dietary sources such as dairy products, meat, eggs, and dark-green leafy vegetables. Inappropriate riboflavin intake can lead to a variety of metabolic disorders and clinical manifestations collectively known as ariboflavinosis [3]. Given its essential role in maintaining human health, the accurate quantification of VB_2_ in biological, pharmaceutical, and food matrices is crucial for nutritional evaluation, quality control, and clinical diagnostics.

The chemical properties of the VB_2_ molecule, such as its water solubility, intrinsic fluorescence, and redox activity, present both opportunities and challenges for analytical determination. Interference from complex matrices, low analyte concentrations, and the need for rapid and cost-effective analysis have driven the development of diverse analytical methods. Classical approaches include fluorometry [4,5,6] and spectrophotometry [7,8,9], which exploit the compound’s intrinsic fluorescence and absorbance properties, respectively. High-performance liquid chromatography (HPLC), often coupled with fluorescence or mass spectrometric detection, remains standard in terms of sensitivity, selectivity, and applicability to complex matrices [10,11,12]. Capillary electrophoresis [13] and enzyme-linked assays [14] have also been employed. While these methods offer high analytical performance, they often require labor-intensive sample preparation, expensive instrumentation, or long analysis times. Electrochemical methods, by contrast, provide attractive alternatives owing to their low cost, high sensitivity, portability, and compatibility with miniaturization for on-site analysis. Among these, voltammetric techniques, such as cyclic voltammetry (CV), differential pulse voltammetry (DPV), and square-wave voltammetry (SWV), have received growing attention for VB_2_ determination, primarily due to its advantageous electrochemical properties imparted by the isoalloxazine ring system [15]. The redox activity of this molecule provides well-defined and reproducible electrochemical signals, thereby enabling sensitive and selective detection across a wide range of electrode platforms, including modified carbon paste electrodes (CPEs) [16,17,18,19,20,21], glassy carbon electrodes (GCEs) [22,23,24,25,26,27], pencil graphite electrodes (PGEs) [28,29,30,31], and screen-printed electrodes (SPEs) [32,33,34,35]. Advances in electrode materials, including carbon nanostructures [36,37], metal nanoparticles [38,39,40], and conducting polymers [41,42,43], have further enhanced the selectivity and sensitivity of voltammetric assays of VB_2_ molecules. These developments have allowed the successful application of voltammetric methods of its determination in diverse matrices, including pharmaceutical formulations, fortified foods, and biological samples, often with minimal pretreatment requirements.

In the area of electrode-modifying nanomaterials, a particularly distinctive position is occupied by zeolites, crystalline aluminosilicates with well-defined pore structures, high surface area, and exceptional ion-exchange capabilities. These materials have attracted significant attention due to their unique ability to selectively host target analytes [44,45] and catalyze redox reactions [46]. Unlike carbon-based nanomaterials, which primarily improve conductivity and provide a large surface, or metal nanoparticles, which offer excellent electrocatalytic activity but may suffer from aggregation or instability, zeolites stand out for their intrinsic molecular recognition, chemical stability, and tunable porosity [47,48]. Nevertheless, the inherently low electrical conductivity of zeolites necessitates their integration with conductive components, most commonly carbon-based materials, to achieve efficient electron transfer. Although graphite [49,50,51] has long been the predominant electron-conducting additive in zeolite-modified electrodes (ZMEs), recent studies increasingly explore carbon black [52], mesoporous carbon [53,54], and graphene oxide [55,56]. The combination of zeolites with advanced carbon-based nanomaterials results in hybrid electrodes with enhanced sensitivity, selectivity, and stability for a wide range of electroanalytical applications.

In this work, a voltammetric sensor was developed by modifying a glassy carbon electrode (GCE) with a nanostructured composite of Ni-exchanged synthetic zeolite Y (NiZY) and carbon nanohorns (CNHs) for the determination of water-soluble B-group vitamins. The synergistic combination of electrocatalytic NiZY and conductive CNHs creates a novel zeolite-modified composite electrode that offers superior electrochemical performance, confirmed by comprehensive morphological, structural, textural, and electrochemical measurements. Optimal electrochemical conditions for VB_2_ determination using NiZY/CNHs-GCEs were established, with selectivity confirmed against potential interferents. Based on CV and DPV studies, the mechanism of redox reaction of VB_2_ molecules on the surface of NiZY/CNHs-GCEs was also proposed. Furthermore, the possibility of VB_2_ analysis in the presence of vitamin B_9_ (VB_9_) was demonstrated and supported by their simultaneous DPV determination in dietary supplements.

## 2. Results and Discussion

### 2.1. Characterization of Electrode Modifiers

**Figure 1:**
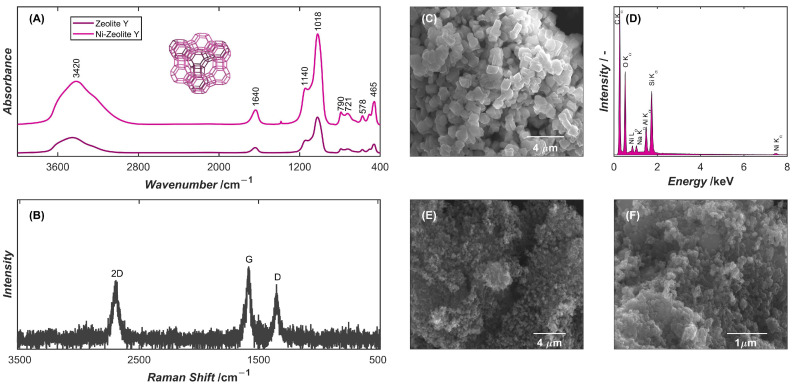
(**A**) FT-IR spectra of zeolite Y before and after ion-exchange with Ni(II) cations in the middle infrared region. (**B**) Raman spectrum of CNHs in the range of 500–3500 cm^−1^. SEM image of (**C**) Ni-zeolite Y and the corresponding (**D**) EDS spectrum, (**E**) CNHs, and (**F**) NiZY/CNH layer deposited on the surface of the GCE.

Structurally, zeolite Y belongs to the faujasite (FAU) framework type, for which [SiO_4_] and [AlO_4_] tetrahedra are arranged into secondary building units (SBUs) in the form of double six-membered rings (D6R). These units link sodalite cages and hexagonal prisms to form large supercages (~12 Å), which are interconnected through a 12-membered ring that defines pore apertures of ~7.4 Å [57]. This structural arrangement results in the presence of characteristic ring vibration bands, which can be clearly distinguished in the FT-IR spectrum of zeolite Y (Figure 1A). An important feature of the FAU framework is the band located at 578 cm^−1^, resulting from D6R vibrations connecting the sodalite cages and defining the overall topology of zeolite Y. The most intense bands appear at about 1140 and 1018 cm^−1^, assigned to asymmetric stretching of Si–O–Si and Si–O–Al bonds, respectively. Weaker bonds at 750–800 cm^−1^ correspond to symmetric stretching vibrations of the tetrahedral units, while the bands in the region of 450–500 cm^−1^ arise from the bending modes of T–O–T (T = Si, Al). Hydroxyl groups and adsorbed water contribute additional bands at 3000–4000 cm^−1^ and ca. 1640 cm^−1^ [58]. Comparison of the FT-IR spectra of zeolite Y before and after Ni(II) ion exchange (Figure 1A) shows that the positions of the aforementioned bands remain essentially unchanged, with only minor variations in their intensity. The observed changes, particularly in the T–O–T and pseudo-lattice regions, reflect the subtle degree of ring deformation caused by incorporation the Ni^2+^ ions into the zeolite Y structure [59].

Since IR spectroscopy is largely insensitive to nonpolar bonds, Raman spectroscopy was employed to characterize the sp^2^ carbon lattice vibrations of CNHs. The Raman spectrum of the CNHs (Figure 1B) exhibited characteristic D (disordered) and G (graphitic) bands with nearly equal scattering strengths located at approximately 1350 and 1580 cm^−1^, respectively. In CNHs, the D band, originating from defects, edges, or lattice distortions, mainly reflects the conical tips and curvature of the nanohorns, while the G band corresponds to the in-plane vibrations of sp^2^ carbon atoms in the graphitic lattice. Additionally, a broad 2D (G′) band is observed around 2700 cm^−1^ reflecting the multilayered and curved nature of the CNH walls [60].

Nitrogen adsorption–desorption analysis (Table 1) indicates that zeolite Y possesses over 2-fold-higher BET surface area and a predominantly microporous structure with an average pore diameter of 1.95 nm, whereas CNHs exhibit limited microporosity, with a relatively large external surface area (284.4 m^2^·g^−1^). These results emphasize that while zeolite Y offers a highly microporous framework, carbon nanohorns provide complementary external surface sites, making their combination particularly promising for the development of sensors with enhanced sensitivity and selectivity.

Morphological characterization of the electrode modifiers shows that zeolite Y (Figure 1C) exhibits a well-defined crystalline structure, with cubic particles characteristic of the FAU framework. The crystals are relatively uniform in size, ranging from 0.5 to 2 µm, and display smooth surfaces with sharp edges, indicative of high crystallinity and controlled synthesis conditions. Concomitantly, the EDS spectrum of zeolite Y after ion exchange with nickel cations (Figure 1D) shows distinct Ni peaks, providing clear evidence of the successful substitution of exchangeable cations within the zeolite structure. Quantitative analysis of nickel in the zeolite, performed on multiple distinct surface points, revealed average Ni atomic concentrations of 0.56 at.%.

The SEM observation of CNHs (Figure 1E) reveals their characteristic tendency to form large aggregates rather than exist as isolated nanostructures. These aggregates appear as spherical or flower-like assemblies composed of numerous nanostructures. As a result of the nanoscale dimensions of CNHs and the dense packing within aggregates, the outlines of individual nanohorns are difficult to distinguish. Finally, the SEM image of the NiZY/CNH composite (Figure 1F) shows the morphological characteristics of both materials. The modifying layer exhibits a heterogeneous microstructure in which the cubic crystals of zeolite Y are uniformly dispersed within a network of CNH aggregates. The CNHs form an interconnected network surrounding the zeolite crystals, resulting in a continuous and stable layer.

### 2.2. Electrochemical Characterization of Modified Electrodes

**Figure 2:**
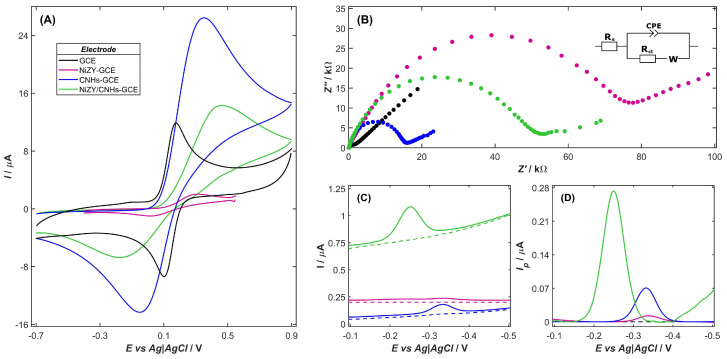
(**A**) The CV voltammograms recorded on bare GCE (black line), NiZY-GCE (pink line), CNHs-GCE (dark blue line), and NiZY/CNHs-GCE (green line) for redox probe of K_4_[Fe(CN)_6_] (1 mmol L^−1^) in 0.1 mol L^−1^ KCl solution (scan rate *v* = 0.05 V s^−1^) and (**B**) corresponding impedance spectra in the form of a Nyquist plot (inset: Randles cell equivalent circuit). (**C**) Comparison of DP curves obtained for 50 µg L^−1^ VB_2_ on the GCE, NiZY-GCE, CNH-GCE, and NiZY/CNHs-GCE (**C**) before and (**D**) after the background subtraction step. Dashed lines denote the background signal. DPV experimental conditions: *E_p_* = −0.1 V, *E_k_* = −0.5 V, *E_s_* = 3 mV, *dE* = 40 mV, *t_s_* = 10 ms, and *t_w_* = 10 ms. Supporting electrolyte: McIlvaine buffer solution (pH 3.4).

The impact of surface modifications on the performance of the developed sensors was examined through CV (Figure 2A) and EIS (Figure 2B) measurements conducted in a 0.1 mol L^−1^ solution containing 1 mmol L^−1^ of the [Fe(CN)_6_]^4−^ model redox probe. To interpret the EIS data, the Nyquist plots were fitted with an equivalent electrical circuit corresponding to the Randles cell (Figure 2B), in which the double-layer capacitance was modeled by a Constant Phase Element (CPE) to reflect the porous and heterogeneous nature of the modified electrodes. On this basis, key parameters such as charge transfer resistance (*R_ct_*), Warburg impedance coefficient (*σ*), electroactive surface area (*A_el_*), double-layer capacitance (*C_dl_*), and the heterogeneous electron-transfer rate constant (*k_s_*) were established and summarized in Table 2. The *R_ct_* and *σ* value for the impedance spectra recorded at the formal potential (*E_f_*) of the redox probe were determined according to Equations (1) and (2):

(1) Rct=RTn2F2Aks·2(DODR)14DO12·CO+DR12·CR

(2) σ=RTn2F2A2·4DO12·CO+DR12·CR

where *R* indicates the universal gas constant (*R* = 8.314 J mol^−1^ K^−1^), *T* is the temperature [K], *F* is the Faraday constant (*F* = 96,485.3 C mol^−1^), *n* is the number of transferred electrons (*n* = 1), *A* is the electroactive surface area of the electrode, *D_O_* and *D_R_* are the diffusion coefficients of the oxidized and reduced forms (7.2 × 10^−6^ cm^2^ s^−1^), respectively, and *C_O_* and *C_R_* are their concentrations in the solution.

The heterogeneous rate constant (*k_s_*) can be determined from the impedance data by taking the ratio of Equations (1) and (2), which gives the expression presented in Equation (3):

(3) $$k_{s}=\frac{\sigma }{R_{ct}}\cdot \sqrt{\frac{D_{O/R}}{2}}$$

where *D_O/R_* indicates the average diffusion coefficient of the oxidized and reduced forms.

As can be seen in Figure 2A, a bare GCE in the presence of a model redox probe shows a reversible electrochemical response characterized by well-defined anodic and cathodic peaks with a peak-to-peak separation (*∆E*) equal to 72 mV (Table 2), indicating efficient electron transfer. At the same time, EIS measurements reveal a high-frequency semicircle corresponding to charge transfer resistance and a low-frequency linear Warburg segment due to diffusion of the redox species. The observed double-layer capacitance value of 24.7 µF cm^−2^ reflects a clean electrode surface that is largely unaffected by adsorption or ion-exchange phenomena.

The electrochemical response of the GCE exhibits notable changes upon modification with the layer containing only zeolite or carbon nanohorns. For the NiZY-GCE, the substantially higher *∆E* (261 mV) compared to the theoretical value indicates a quasi-reversible [Fe(CN)_6_]^3−^/^4−^ redox process, arising from the insulating and porous nature of the zeolite framework, which restricts charge transfer between the redox probe and the electrode. The pronounced decrease in both the electroactive surface area (4.2 mm^2^) and the peak current (1.0 µA) further indicates the limited number of active sites for the redox probe. These observations are consistent with the high charge-transfer resistance (*R_ct_* = 70.7 kΩ) and the low heterogeneous rate constant (*k_s_* = 1.6 × 10^−6^ cm s^−1^), both of which confirm that electron transfer is strongly hindered by the zeolite film. Additionally, the elevated Warburg coefficient indicates diffusion limitations within the zeolite micropores. Together, these factors confirm that the electrochemical response of the NiZY-GCE is significantly influenced by the porous structural and resistive properties of zeolites. In the case of the CNHs-modified GCE, a notable asymmetry between the anodic and cathodic peak currents, along with the large peak separation (∆*E* = 360 mV) and the high *I_pa_*/*I_pc_* ratio (1.2), indicates a quasi-reversible electron-transfer behavior. The excellent electrical conductivity and the high surface area of CNHs lead to a substantial increase in the anodic peak current (*I_pa_* = 21.0 µA) and the electroactive surface area (*A_el_* = 9.7 mm^2^), thereby improving the accessibility of the redox probe to active sites. In contrast to an insulating zeolite-containing film, the CNH layer facilitates electron transfer, as evidenced by the relatively low *R_ct_* and higher *k_s_* values, reflecting faster electron-transfer kinetics. Moreover, the conical morphology of CNHs creates interconnected pathways that enhance diffusion of the redox probe, thereby reducing mass-transfer limitations, as indicated by the smaller Warburg coefficient compared to the NiZY-GCE.

Ultimately, the NiZY/CNHs-GCE exhibits enhanced electrochemical performance due to the complementary properties of zeolite and carbon nanohorns. The zeolite provides a well-structured microporous framework that can pre-concentrate the redox probe, facilitating its interaction with the electrode surface. At the same time, the CNHs offer high conductivity and form an interconnected pathway network that enables efficient electron transfer throughout the electrode. This synergy results in an increased electroactive surface area (*A_el_* = 10.0 mm^2^) and reduced mass-transfer limitations (*σ* = 2.8 kΩ·s^−1/2^). By combining these materials, the composite electrode achieves a favorable balance between high surface area, number of active sites, and efficient electron-transfer pathways, illustrating the advantage of integrating zeolites and carbon nanohorns for the improved electrochemical performance of NiZY-CNHs.

The favorable electrochemical properties of the NiZY/CNHs-GCE can be further demonstrated by its improved analytical performance in VB_2_ determination, as confirmed by the enhanced current response compared to the bare GCE and electrodes modified with each component individually (Figure 2C). For the bare GCE, which served as the substrate for the deposition of the modifying layer, the DPV signal corresponding to the reduction of 50 µg L^−1^ VB_2_ was highly unrepeatable due to pronounced adsorption of the vitamin on the unmodified electrode surface. This poor reproducibility makes the GCE unsuitable for reliable analysis, emphasizing the need to modify its surface to achieve accurate electrochemical detection of VB_2_.

The deposition of the layer containing only zeolite nanoparticles led to very low electrochemical activity of NiZY-GCE toward VB_2_ molecules. This is especially apparent after background subtraction, which reveals only a minimal current response of NiZY-modified GCE (Figure 2D), likely due to the insulating nature and microporous structure of the zeolite Y. In contrast, modification of the GCE with a CNHs-containing layer allowed the recording of a stable and reproducible reduction signal for VB_2_, distinguished by its reversible character and well-defined shape. However, the corresponding peak current is nearly four times lower than that obtained for the NiZY/CNHs-GCE, underscoring the superior performance of the composite electrode. Furthermore, the observed shift of the peak potential toward a less negative value, from −0.332 V and −0.339 V for the CNHs-GCE and NiZY-GCE, respectively, to −0.249 V for the NiZY/CNHs suggests a catalytic effect, indicating that the composite facilitates the analyte redox process and improves reaction kinetics by lowering overpotential for VB_2_ reduction. Moreover, the DPV results (Figure 2C) show a clear increase in the capacitive background current in the following order: bare GCE < CNHs-GCE < NiZY-GCE < NiZY/CNHs-GCE. The higher background signal for NiZY-GCE compared to CNHs-GCE indicates a strong adsorption of analyte molecules by the zeolite layer, even though zeolite is electrically insulating. The highest response for the NiZY/CNHs-GCE results from the synergistic effect of the conductive CNHs and the high adsorption capacity of NiZY. However, the *C_dl_* values from EIS (Table 2) decrease in the opposite order, which is caused by the strongly heterogeneous and porous nature of the modified layers. This interpretation is supported by the DPV results, which confirm that the actual electroactive area increases despite the lower apparent *C_dl_* from EIS. These differences highlight the advantageous role of both composite components: while CNHs contribute high conductivity and efficient charge transport, the addition of Ni-exchanged zeolite introduces a microporous framework capable of pre-concentrating VB_2_ molecules near the electrode surface, enhancing both the number of active sites and the kinetics of electron transfer, thereby delivering superior electrochemical performance for the composite electrode.

### 2.3. Investigation of the Redox Behavior of VB_2_ on the NiZY/CNHs-GCE

**Figure 3:**
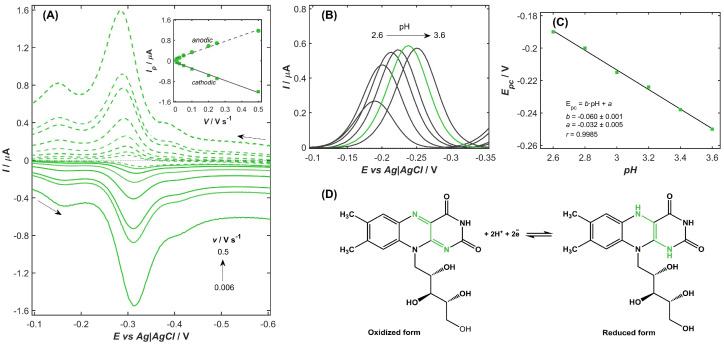
(**A**) CV curves registered in McIlvaine buffer solutions (pH 3.4) in the presence of 1 mg L^−1^ of VB_2_ on the NiZY/CNHs-GCE with a scan rate from 0.006 to 0.5 V s^−1^ (arrows—scan direction; solid lines—the cathodic scan; dashed lines—the anodic scan). Inset: the relationships between the peak current *Ip* and the scan rate *v*. (**B**) DPVs recorded for the reduction of 0.2 mg L^−1^ VB_2_ in McIlvaine buffer solution at pH from 2.6 to 3.6 after background subtraction. (**C**) The cathodic peak potential (*E_pc_*) as a function of the pH of the supporting electrolyte. (**D**) The proposed mechanism of the VB_2_ redox reaction.

The electrochemical behavior of VB_2_ molecules on the NiZY/CNHs-GCE was systematically investigated using CV and DPV measurements. VB_2_ at a concentration of 1 mg L^−1^ was subjected to CV analysis at scan rates ranging from 0.006 to 0.1 V s^−1^ (Figure 3A). The recorded voltammograms displayed well-defined anodic and cathodic peaks, characteristic of a reversible redox couple corresponding to the electron-transfer reaction of VB_2_. The near-unity ratio of anodic to cathodic peak currents (*I_pa_/I_pc_* = 1.01) and the unchanged peak positions over the range of scan rate indicate that the electron-transfer process is fully reversible and kinetically facile under the experimental conditions. The dependence of peak currents on scan rate provided further mechanistic insight. The obtained linear relationship between the peak currents and scan rate (inset in Figure 3A), rather than the square root of scan rate, indicated that the VB_2_ redox reaction is adsorption-controlled. For accurate determination of the number of electrons (*n*) involved in the electrochemical redox reaction of VB_2_ molecules in adsorption-controlled electrochemical systems, the difference between the cathodic peak potential (*E_ca_*) and the potential corresponding to half of the peak current (*E_ca/2_*) was carefully measured and found to be 0.032 V, according to Equation (4) [61]:

(4) $$\left|E_{pc}-E_{pc/2}\right|=\frac{0.0565}{n}\;\;\;\;\;\;[V]$$

The value corresponding to an electron-transfer number was calculated to be 1.7, indicating that the electrochemical redox reaction of VB_2_ occurs through a two-electron process.

The redox behavior of VB_2_ molecules was further analyzed in supporting electrolyte of varying pH values. For this purpose, DPV measurements were performed in a series of McIlvaine buffer solutions with pH values ranging from 2.6 to 3.6, and the corresponding signals of VB_2_ at a concentration of 0.2 mg L^−1^ were recorded for each solution (Figure 3B). The voltammograms revealed that both anodic and cathodic peak potentials shifted negatively with increasing pH, indicating the participation of protons in the electrochemical redox process. A linear relationship was observed between the VB_2_ cathodic peak potential and pH (Figure 3C) with a slope of (−0.060 ± 0.001) V per unit change in pH, close to theoretical value of 0.059 V pH^−1^, suggesting that the number of protons involved in the reaction is equal to the number of electrons transferred.

The results of this study demonstrate that the electrochemical redox reaction of VB_2_ molecules at the NiZY/CNHs-modified GCE proceeds through adsorption-controlled kinetics involving the exchange of two electrons and two protons, as illustrated in Figure 3D. The reversible reduction occurs at the isoalloxazine ring, where electron transfer converts VB_2_ between its oxidized quinone-like state and the reduced hydroquinone-like form. This behavior is consistent with previously reported mechanisms for flavin systems, confirming the proton-coupled, two-electron nature of the VB_2_ redox process [20,27,31,34].

### 2.4. Optimization of the Experimental Conditions

As shown in Figure 3C, the peak current of VB_2_ molecules depends on the pH of the McIlvaine buffer solution, with the highest response observed at pH 3.4 (green line), which was selected as the optimal condition for subsequent measurements. Concurrently, to achieve the most sensitive and accurate detection of VB_2_, the key parameters of the DPV technique were systematically optimized. These included step potential (*E*_s_), which was changed from 1 to 6 mV; pulse amplitude (*dE*), which was varied from 10 to 60 mV (both positive and negative modes); and current sampling time (*t_s_*) and waiting time (*t_w_*), which were modified from 5 to 40 ms. Each parameter was individually varied to assess its effect on the VB_2_ reduction signal in terms of peak height, shape, symmetry, and potential. In consequence, the optimal conditions for DPV determination of VB_2_ were chosen as follows: *E_s_* = 3 mV, *dE* = 40 mV, *t_s_* = 10 ms, and *t_w_* = 10 ms.

### 2.5. Analytical Performance

**Figure 4:**
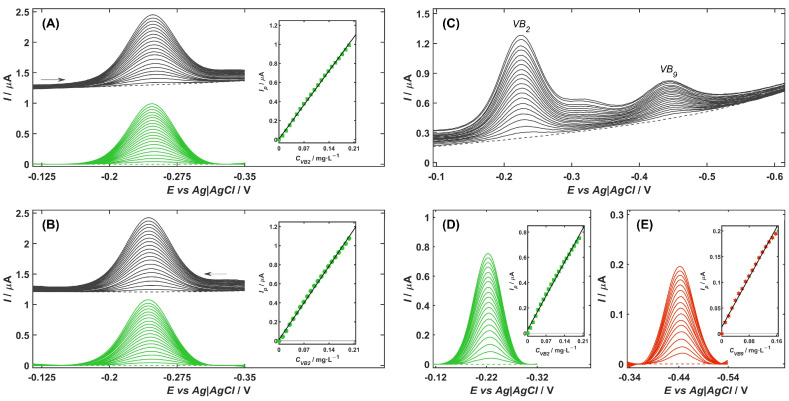
(**A**) Cathodic and (**B**) anodic DPV calibration voltammogram (scan direction indicated by arrow) registered on the NiZY/CNH-GCE for the increasing concentration of VB_2_ from 0 (dashed line) to 0.2 mg L^−1^ (black lines) with curves after subtracted background current (green lines). Inset: corresponding calibration plots. (**C**) DP cathodic voltammograms of mixture solution containing VB_2_ and VB_9_ at the concentration from 0 to 0.2 mg L^−1^. (**D**) VB_2_ and (**E**) VB_9_ curves obtained after baseline correction with corresponding calibration graphs. Experimental conditions as described in Section 3.3.3.

The sensitivity of VB_2_ determination on the NiZY/CNHs-GCE in McIlvaine buffer solution (pH 3.4) was evaluated under optimal conditions by DPV measurements. Each voltammogram, recorded at different concentrations of VB_2_ molecules, was background-corrected by subtracting the signal obtained in the supporting electrolyte without the depolarizer (dashed line in Figure 4), resulting in the color-marked curves. Linearity was assessed by considering both the cathodic and anodic peak currents, and the corresponding calibration plots of peak current versus VB_2_ concentration were constructed and shown in Figure 4A,B, respectively. In addition, the analytical performance of the VB_2_ sensor was also tested in the presence of vitamin B_9_ (VB_9_) to demonstrate the possibility of their simultaneous determination. DPV measurements were conducted under the same parameters as in the individual experiment, with the exception of the supporting electrolyte, which was McIlvaine buffer at pH 3.0. The cathodic responses of VB_2_ and VB_9_ molecules in the mixture were investigated when the concentrations of both vitamins were simultaneously varied in the range from 0.01 to 0.2 mg L^−1^. The resulting DVP curves, along with the corresponding calibration graphs, are presented in Figure 4C–E. The calculated calibration curve coefficients and analytical characteristics for the individual and simultaneous determination of VB_2_ and VB_9_ molecules are summarized in Table 3.

For individual determination of VB_2_, both cathodic (Figure 4A) and anodic (Figure 4B) peaks were observed at similar potentials of approximately −0.25 V. The well-developed and symmetric nature of the obtained signals confirms the reversible redox behavior of riboflavin on the NiZY/CNHs-GCE surface. In both directions, an excellent linear correlation was achieved between the peak current and the VB_2_ concentration in the range of 0.01 to 0.2 mg L^−1^, highlighting the reliable quantitative performance of the sensor. The limit of detection (*LOD*) and the limit of quantification (*LOQ*) were estimated using the equations *LOD* = 3.3·*SD*/*b* and *LOQ* = 10·*SD*/*b*, where *SD* is the standard deviation of the *y*-intercept of the regression line and *b* is the slope of the calibration curve. The results obtained for both anodic and cathodic responses were comparable (Table 3), with *LOD* values in the nanomolar range, demonstrating the suitability of the NiZY/CNHs-GCE for trace-level determination of VB_2_ molecules.

In the simultaneous determination of VB_2_ and VB_9_, a significant separation between their reduction peak potentials was observed (Figure 4C), allowing for distinct and interference-free electrochemical signals for each analyte. In the mixture solution containing both vitamins, the sensor exhibited a wide and linear range, extending from 0.01 to 0.20 mg L^−1^ for VB_2_ and from 0.01 to 0.16 mg L^−1^ for VB_9_. Although the presence of VB_9_ caused a slight reduction in the sensitivity to VB_2_ determination, this effect did not significantly compromise the overall analytical performance of the electrode. In fact, despite this signal attenuation, the proposed method achieved an *LOD* in the nanomolar range for both vitamins, underlining the high sensitivity of the NiZY/CNHs-GCE. It is worth emphasizing that the ability to simultaneously quantify VB_2_ and VB_9_ represents a significant analytical advantage of the proposed sensor, especially for applications involving complex biological or pharmaceutical matrices, where both vitamins may coexist at trace levels.

As shown in Table 4, various voltammetric sensors have been developed for the electrochemical determination of VB_2_ molecules, employing different electrode modification strategies to enhance analytical performance in terms of sensitivity, selectivity, and detection limits. Although they provide an overall analytical advantage, these modification strategies are limited by inherent material challenges. For example, carbon-based nanomaterials often require chemical activation or surface functionalization and tend to agglomeration, which can reduce reproducibility and long-term stability. Metal nanoparticles may suffer from instability, high reactivity, potential toxicity, safety concerns, and complex synthesis procedures involving hazardous reagents and high energy input. Conducting polymer can exhibit poor mechanical stability, dopant-dependent signal drift, non-uniform coatings, and limited stability in extreme conditions [62].

In contrast, NiZY/CNHs-GCE provides a simple, robust, and cost-effective alternative for the electrochemical sensing of VB_2_ molecules. The synergistic combination of zeolite Y and carbon nanohorns endows a uniquely structured electrode surface with a high surface area and abundant active sites that facilitate efficient electron transfer. Zeolite Y contributes excellent ion-exchange capacity and chemical stability, providing a well-defined microenvironment for the dispersion of Ni^2+^-based catalytic sites and enhanced selectivity toward VB_2_ molecules. Meanwhile, CNHs offer outstanding conductivity and mechanical strength without requiring any chemical pretreatment, which constitutes a distinct advantage over graphene or carbon nanotubes. This NiZY/CNHs-GCE not only achieves comparable or superior analytical performance to sensors based on noble metals or multifunctional polymers but does so with significantly lower cost, simpler preparation, and higher environmental safety. The fabrication procedure is straightforward, requiring only basic laboratory equipment and avoiding any hazardous reagents or sophisticated apparatus. The resulting suspension remains stable for days, enabling the preparation of multiple electrodes with consistent performance. Furthermore, sensors fabricated by a simple drop-casting method exhibit exceptional reproducibility, long-term operational stability, and mechanical durability, maintaining their electrochemical response over hundreds of measurements without special storage conditions. The last statement is further supported by the results of stability and reproducibility studies, which showed that ten consecutive voltammetric scans at VB_2_ concentrations of 50 µg L^−1^ and 100 µg L^−1^ recorded under identical conditions resulted in relative standard deviation (*RSD*) values of 2.4% and 1.3%, respectively. These results confirmed excellent short-term stability and repeatability with a consistent signal response during successive measurements. On the other hand, after 7, 14, and 21 days of sensor storage under laboratory conditions, the peak current for both VB_2_ concentrations remained above 87% of the initial value, indicating minimal signal drift and confirming robust long-term stability of NiZY/CNHs-GCE. Reproducibility was further verified by comparing the responses of five independently prepared electrodes under the same conditions, resulting in an RSD of 8.6%, demonstrating reliable electrode fabrication and uniform analytical performance.

In summary, the NiZY/CNHs-GCE combines the structural robustness of zeolites, the electrochemical efficiency of carbon nanostructures, and the cost-effectiveness of scalable fabrication. This unique combination positions it as a highly competitive alternative to more complex nanocomposite systems, offering an ideal balance between analytical performance, environmental sustainability, and practical usability for routine electrochemical applications.

### 2.6. Selectivity

The selectivity of NiZY/CNH-GCE was also evaluated by testing its response to VB_2_ in the presence of potentially interfering species representing other vitamins, common biomolecules, pharmaceutical excipients, and surface-active compounds. For this purpose, the solution containing VB_2_ at a concentration of 50 µg L^−1^ was combined with a 4- to 200-fold excess of vitamin B_6_ (VB_6_) and vitamin C (VC), a 20- to 1000-times excess of glucose and caffeine, and 10- to 200-times excess of surface-active compounds, including sodium dodecyl sulphate (SDS), cetrimonium bromide (CTAB), and Triton-X100. The effect of common excipients of dietary supplements (titanium dioxide, starch, magnesium stearate, and cellulose) was tested individually by adding an appropriate weighed amount of 2 to 10 mg of each substance directly to the voltammetric cell. DPV measurements conducted in both the absence and in the presence of each individual species were used to evaluate changes in VB_2_ peak current, potential, and shape, thus assessing possible interference effects. Tolerance limits were defined as the highest interferent concentration that causes no more than a ±10% change in peak current.

Of the vitamins evaluated, VB_6_ has a pronounced effect on the VB_2_ reduction signal, increasing the peak current by 50% at 200-fold excess, while the addition of vitamin C did not cause significant change in the peak current, shape, and position. The observed enhancement of the VB_2_ peak current in the presence of the highest excess of VB_6_ may be attributed to effects of adsorption or surface activation. Specifically, VB_6_ molecules may undergo a partial co-adsorption, which locally enhances the concentration of VB_2_ near the electrode surface and/or modify its physicochemical properties, such as surface roughness or hydrophilicity, thus improving the accessibility and electron transfer efficiency of VB_2_. In the case of common biomolecules, only caffeine exhibits an interference effect causing enhancement in the voltammetric response of NiZY/CNHs-GCE to 120% of the initial value of VB_2_ peak current at 1000-fold excess. A more significant impact is related to surface-active compounds, i.e., Triton X-100 (nonionic surfactant) and CTAB (cationic surfactant), the presence of which in the 200-fold excess is associated with a 22% and 50% decrease in VB_2_ reduction signal, respectively. Among dietary supplement excipients, a slight signal increase (less than 17%) is observed for the highest tested amount of magnesium stearate added to the measurement cell. On the other hand, cellulose demonstrated a suppressive effect on the VB_2_ signal, reducing it to approximately 87% of the initial value, which can be connected with the solid phase present in the supporting electrolyte that limits the electrode reaction. Nevertheless, minimizing the interference effect from tablet excipients can be achieved by implementation of appropriate sample preparation strategies (e.g., solid particulate filtration).

The results of the selectivity assessment demonstrate that although some compounds can influence the VB_2_ response at very high excess, the proposed NiZY/CNHs-GCE provides reliable performance and selectivity under typical sample conditions.

### 2.7. Analytical Application

**Figure 5:**
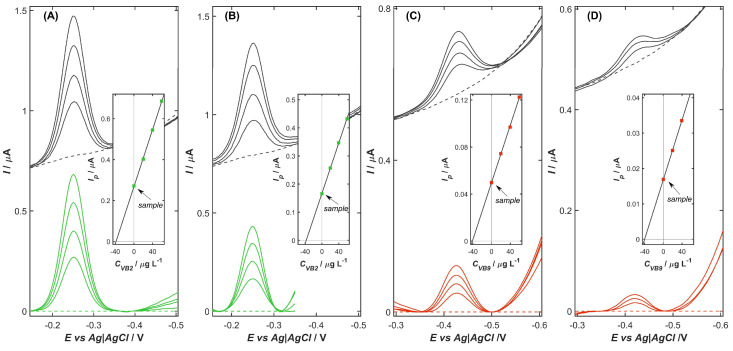
Experimental voltammograms (black) and background-corrected curves (color marked) for the analysis of (**A**) *Apteo Witamina B*_2_, (**B**) *Panawit Witamina B*_2_, (**C**) *Olimp Labs Kwas foliowy*, and (**D**) *ActiFolin*. Inset: corresponding calibration plots. Experimental conditions as described in Section 3.3.3.

The NiZY/CNHs-GCE sensor was further evaluated for its applicability in the analysis of commercial vitamin dietary supplements (Figure 5) containing VB_2_ and VB_9_. Sample preparation was carried out according to the procedure described in Section 3.3.2 and analyzed using the standard addition method, which allows for accurate quantification in complex matrices and minimizes matrix-related interferences. The results of the measurements are summarized in Table 5.

For both vitamins, a well-defined peak corresponding to their reduction was observed on the surface of the NiZY/CNHs-GCE. Furthermore, peak currents increased linearly with each addition of the standard, demonstrating the quantitative response of the sensor for both VB_2_ and VB_9_ molecules in complex sample matrices. The measured vitamin content (Table 5) corresponds closely to the manufacturer’s declared values, with a relative error (*RE*) ranging from −6.2% to 2.7%, clearly indicating that the sensor provides accurate determinations of both analytes. At the same time, the excellent precision with relative standard deviation (*RSD*) below 2.1% confirms reliable and precise quantification in complex supplement matrices. Importantly, all measured values fall within acceptable tolerance limits for dietary supplements, further validating the suitability of the method for routine quality control of dietary supplements.

## 3. Materials and Methods

### 3.1. Chemicals and Solutions

Zeolite Y (ZY) in the sodium form was acquired from Thermo Scientific (Waltham, MA, USA) (Alfa Aesar). Carbon nanohorns, as grown (CNHs), and polystyrene (average M_w_~35,000) were purchased from Sigma-Aldrich (St. Louis, MO, USA). Organic solvent, i.e., dichloromethane (ACS Reagent), and an acetone solution suitable for HPLC (≥99.9%) were obtained from Honeywell Research Chemicals (Seelze, Germany) and Avantor Performance Materials Poland S.A. (Gliwice, Poland), respectively. Nickel(II) nitrate hexahydrate, 98%, vitamin B_2_ (riboflavin, Secondary Pharmaceutical Standard), and vitamin B_9_ (folic acid, meeting USP testing specifications) were acquired from Sigma-Aldrich. The standard stock solution of vitamin B_2_ (VB_2_), as well as vitamin B_9_ (VB_9_) at a concentration of 1000 mg L^−1^ with the addition of 0.02 mol L^−1^ NaOH, was prepared weekly and stored at 4 °C under light-protected conditions. Working solutions of lower concentrations were prepared daily by diluting the stock solutions of VB_2_ and VB_9_ with double-distilled water. The supporting electrolyte in the form of citrate-phosphate buffer (McIlvaine buffer) was prepared by mixing appropriate volumes of 0.1 mol L^−1^ citric acid (Avantor Performance Materials Poland S.A.) with 0.2 mol L^−1^ Na_2_HPO_4_ (Avantor Performance Materials Poland S.A.) in order to obtain a solution with a pH value in the range of 2.6 to 3.6. Tested interference compounds, i.e., vitamin B_6_ (pyridoxine, ≥98%), L-ascorbic acid (ACS reagent grade, ≥99%), caffeine, Triton X-100, sodium dodecyl sulfate (SDS), titanium dioxide, starch, cellulose, and magnesium stearate were acquired from Sigma-Aldrich, whereas glucose and cetrimonium bromide (CTAB) were purchased from Avantor Performance Materials Poland S.A. All reagents were of analytical grade and used without further purification.

### 3.2. Instrumentation

The infrared spectrum of zeolite Y was recorded in the mid-infrared region (4000–400 cm^−1^) using a Vertex 70v FT-IR spectrometer (Bruker, Billerica, MA, USA) with KBr pellets (256 scans, 4 cm^−1^ resolution). In turn, Raman spectrum of CNHs was collected using a WITec Alpha 300 M+ (WITec GmbH, Ulm, Germany) with a 600 groove mm^−1^ grating and a 532 nm laser with adjusted power to prevent sample degradation. A Zeiss Epiplan Neofluar (Carl Zeiss AG, Oberkochen, Germany) 20× long-working-distance objective (laser spot ~0.5 μm) was used. XY maps (40 × 40 μm^2^, 0.5 μm step size, 0.5 s per spectrum) were acquired, and data were processed using WITec Project Five 5.3 PRO software. The texture features of ZY and CNHs were established by multipoint N_2_ adsorption/desorption at 77 K using an ASAP 2010 (Micromeritics, Norcross, GA, USA). The samples were degassed at 623 K for 24 h prior to analysis. The specific surface area (SSA) and pore size distribution were calculated using the Brunauer–Emmett–Teller (BET) and Barrett–Joyner–Halenda (BJH) methods, respectively. The morphology of ZY, CNHs, and the modifying layer was examined using a Thermo Scientific Scios 2 DualBeam, an ultra-high-resolution analytical-focused ion beam scanning electron microscope (FIB-SEM). Elemental analysis of ZY before and after Ni(II) cation exchange was performed using an energy-dispersive X-ray spectroscopy (EDS) attachment.

Voltammetric measurements were performed using an M161 electrochemical analyzer with an M164D electrode stand (mtm-anko, Kraków, Poland) and EALab 2.1 software. A 10 mL three-electrode cell consisted of a bare or modified GCE (MF-2012, φ = 3 mm, BASi, Bioanalytical Systems, Inc., West Lafayette, IN, USA) as the working electrode, a double-junction Ag | AgCl | 3 M KCl reference electrode (MINERAL, Gliwice, Poland), and a Pt wire auxiliary electrode. Electrochemical impedance spectroscopy (EIS) was performed using a μAUTOLAB III analyzer (EcoChemie, Utrecht, The Netherlands) with NOVA 2.0 software for data acquisition. The solutions were stirred at ~200 rpm with a Teflon^®^-coated magnetic stir bar (Kartell, Noviglio, Italy) and deoxygenated by argon purging before measurements in the negative potential range. The pH value of McIlvaine buffer was adjusted using a SevenCompact S210 pH meter (Mettler Toledo, Greifensee, Switzerland).

### 3.3. Procedures

#### 3.3.1. Zeolite Y Modification and NiZY/CNHs Electrode Fabrication

To enhance electrocatalytic activity, zeolite Y was converted to its nickel form (NiZY) by an ion exchange process carried out in the following steps: (1) 0.5 g of ZY was treated with 5 mL of 0.2 M Ni(NO_3_)_2_·6H_2_O solution and shaken (2500 rpm) for 24 h at 25 °C; (2) after centrifuging at 13,500 rpm (10 min), the procedure was repeated twice, each time using a fresh Ni(II) solution; (3) the resulting material was thoroughly rinsed with double-distilled water to remove surface adhering salts and dried at 40 °C for 24 h in a drying oven. The incorporation of nickel ions in the zeolite Y structure was confirmed by SEM/EDS analysis.

To prepare the modified sensor, the CNHs without any pretreatment and NiZY were ground together in an agate mortar in a 1:1 *w*/*w* ratio, after which 10 mg of homogenized mixture was transferred to an Eppendorf tube and suspended in 500 µL of organic solvents, i.e., acetone and dichloromethane (2:3 *v*/*v* ratio), containing 5 mg of polystyrene and homogenized for 30 min (1800 rpm) using a Vortex Multi Speed MSV-3500 (BioSan, Riga, Latvia). In the next step, 10 μL of the modifying suspension was carefully dropped onto a polished surface of GCE (0.3 μm alumina, Buehler Micropolish II, Lake Bluff, IL, USA) and left for the solvent to evaporate under laboratory conditions for 24 h. Thereafter, the obtained NiZY/CNHs-GCE was stored under ambient conditions and used directly in measurements without any further activation. Following the same approaches, single-component electrodes containing 10 mg of either NiZY or CNHs were fabricated, resulting in NiZY-GCE and CNHs-GCE, respectively.

#### 3.3.2. Sample Preparation and Analysis

Dietary supplements in the form of tablets containing riboflavin *Apteo Witamina B*_2_ (3 mg VB_2_/tablet; Synoptis Pharma Sp. z o.o., Warsaw, Poland) and *Panawit Witamina B*_2_ (10 mg VB_2_/tablet; PANAWIT Sp. z o.o., Kraków, Poland), as well as folic-acid-containing dietary supplements, *Olimp Labs Kwas foliowy* (400 µg VB_9_/tablet; OLIMP LABORATORIES Sp. z o.o., Dębica, Poland) and *ActiFolin* (800 µg VB_9_/tablet; POLSKI LEK Sp. z o.o., Warsaw, Poland), were purchased from a local pharmacy. For their voltammetric analysis, a stock solution of each vitamin (10 mg L^−1^) was prepared by weighing and grinding five tablets of each brand in an agate mortar. An appropriate portion of the resulting powder was transferred to a 100 mL (VB_2_) or 25 mL (VB_9_) amber gold volumetric flask and dissolved in double-distilled water. The solutions were then homogenized by sonication for 15 min and filtered through a 0.22 μm Mixed Cellulose Ester (MCE) syringe filter (Alchem Grupa, Nowa Sól, Poland) to remove insoluble excipients.

For the DPV analysis of VB_2_, 20 µL of corresponding dietary supplement solution was added to 5 mL of McIlvaine buffer solution (pH 3.4), and measurements were carried out using the standard addition method. At each addition step, four consecutive DPV scans were recorded, followed by background correction, and the mean peak current was determined. The VB_2_ content in the analyzed tablets was calculated from the extrapolated intercept of the resulting calibration plot. Vitamin B_9_-containg dietary supplements were analyzed in the same manner, except that McIlvaine buffer at pH 2.8 was used.

#### 3.3.3. Standard Electrochemical Procedure

The modified electrodes were electrochemically characterized by electrochemical impedance spectroscopy (EIS) and cyclic voltammetry (CV) measurements performed in a 0.1 mol L^−1^ KCl solution containing 1 mmol L^−1^ of the reversible redox probe in the form of [Fe(CN)_6_]^4−^. Sinusoidal signals of frequency ranging from 100 kHz to 25 mHz and 10 mV amplitude, superimposed on the formal potential of K_4_[Fe(CN)_6_], were used in EIS measurements. Electrochemical figures of merit were obtained by applying an equivalent electrical circuit (EEC) model to fit the experimentally recorded impedance spectra.

To establish the electrochemical behavior of VB_2_ on the surface of the NiZY/CNHs-GCE, both CV and DPV analysis were applied. Cyclic voltammograms were recorded in McIlvaine buffer at pH 3.4 containing 1 mg L^−1^ of VB_2_, in the potential range of −0.1 to −0.6 V with a scan rate (*v*) that changed from 0.006 to 0.5 V s^−1^. In turn, DPV measurements were conducted in McIlvaine buffer solutions over a pH range of 2.6 to 3.6 in the presence of 0.2 mg L^−1^ VB_2_. The analytical performance and quantitative analysis of VB_2_ were performed using DPV in both anodic and cathodic directions. Measurements were carried out under optimized experimental conditions, which included McIlvaine buffer at pH 3.4, step potential (*E_s_*) = 3 mV, pulse amplitude (*dE*) = 40 mV, waiting time (*t_w_*) = 10 ms, and current sampling time (*t_s_*) = 10 ms. For VB_2_ and VB_9_ simultaneous determination, the cathodic response of NiZY/CNHs-GCE was recorded in McIlvaine buffer at pH 3.0 under the same optimized instrumental parameters of the DPV technique. All measurements were performed in a 5 mL electrochemical cell, recording at least four consecutive voltammograms, and the background-corrected peak currents were averaged for the evaluation of the obtained data.

## 4. Conclusions

In this work, a novel zeolite-modified electrochemical sensor was developed by modifying the surface of GCEs with a composite of Ni-exchanged zeolite Y and carbon nanohorns, The resulting NiZY/CNHs-GCE demonstrated markedly improved electrochemical activity toward the reduction of VB_2_ molecules, arising from the synergistic effect of both materials, which provides high surface area, enhanced probe accessibility, and efficient electron-transfer pathways. Morphological, structural, and voltammetric analyses confirmed the effective integration of NiZY and CNHs, resulting in the formation of an active sensing layer with favorable electrochemical parameters. As indicated by CV and DPV measurements, the redox reaction of VB_2_ molecules at the NiZY/CNHs-GCE follows adsorption-controlled kinetics, involving the transfer of two electrons and two protons.

The proposed DPV-based voltammetric method for the sensitive quantification of VB_2_ molecules allows us to achieve *LOD* values in the nanomolar range (8.6 × 10^−9^ mol L^−1^), highlighting its suitability for trace analysis. At the same time, the proposed NiZY/CNHs-GCE offers a notable analytical advantage by enabling the simultaneous quantification of VB_2_ and VB_9_, which is particularly valuable in complex biological or pharmaceutical matrices where both vitamins may be present at low concentrations. The close correspondence of the measured vitamin levels with the declared values, expressed as an *RE* higher than −6.2%, confirms the accuracy determinations of both analytes. The findings highlight the promising applicability of the developed sensor in routine quality control of dietary supplements, providing a reliable and accurate platform for trace-level vitamin analysis.

## Figures and Tables

**Table 1:** Textural parameters of employed zeolite Y and CNH.

Material	Surface Area [m^2^ g^−1^]			Pore Volume [cm^3^ g^−1^]		Average Pore Diameter [nm]
	*S*_*BET*_	*S*_*micro*_	*S*_*ext*_	*V*_*micro+mezo*_	*V*_*micro*_	
Zeolite Y	1001.81	992.65	9.16	0.511	0.461	1.95
Carbon nanohorns	460.65	176.27	284.38	0.152	0.075	0.14

*S_BET_*—BET surface area; *S_micro_*—micropore area; *S_ext_*—external surface area; *V_micro+mezo_*—micropore + mezopore volume; *V_micro_*—micropore volume.

**Table 2:** The comparison of electrochemical properties of tested electrodes achieved from CV and EIS measurements.

Parameter	Unit	Working Electrode			
		GCE	NiZY-GCE	CNHs-GCE	NiZY/CNHs-GCE
Formal potential, *E_f_*	mV	138	164	152	160
Peak separation, ∆*E*	mV	72	261	360	536
Anodic peak current, *I_pa_*	µA	9.6	1.0	21.0	9.8
Anodic to cathodic peak current, *I_pa_/I_pc_*	-	0.99	0.9	1.2	1.4
Charge-transfer resistance, *R_ct_*	kΩ	1.4	70.7	12.2	48.0
Warburg coefficient, *σ*	kΩ s^−1/2^	3.15	6.70	2.90	2.80
Electroactive surface area, *A_el_*	mm^2^	8.9	4.2	9.7	10.0
Double-layer capacitance, *C_dl_*	µF cm^−2^	24.7	15.7	6.3	4.2
Heterogeneous rate constant, *k_s_*	m s^−1^	4.3 × 10^−^^5^	1.6 × 10^−6^	4.5 × 10^−6^	1.4 × 10^−6^

**Table 3:** Summary of analytical parameters for VB_2_ in individual and simultaneous determination with VB_9_.

Parameter	Unit	VB_2_		VB_2_ in the Presence of VB_9_	VB_9_ in the Presence of VB_2_
		Cathodic	Anodic		
Linear range	mg L^−1^	0.01–0.2	0.01–0.2	0.01–0.2	0.01–0.16
Intercept *a*	µA	0.008 ± 0.005	0.014 ± 0.006	0.027 ± 0.006	0.012 ± 0.002
Slope *b*	µA L mg^−1^	5.10 ± 0.04	5.51 ± 0.05	3.82 ± 0.06	1.21 ± 0.03
*r*	-	0.9993	0.9991	0.9979	0.9964
LOD	µg L^−1^ (nM)	3.2 (8.6)	3.6 (9.5)	5.2 (13.8)	5.4 (12.3)
LOQ	µg L^−1^ (nM)	9.8 (26.1)	10.9 (28.9)	15.7 (41.8)	16.5 (37.3)
Figure		(A)	(B)	(D)	(E)

**Table 4:** Analytical performances of voltammetric methods for determination of VB_2_ molecules.

Electrode	Technique	Linear Range [µmol L^−1^]	LOD [nmol L^−1^]	Samples	Ref.
^1^ Co^2+^-Y/CPE	CV	1.7–34	710	Multivitamin tablet	[16]
^2^ MnTPP/CPE	DPV	0.01–10	8.0	Food, pharmaceuticals	[17]
^3^ MnO_2_/CPE	DPV	0.02–9	15	Tablet	[18]
^4^ ShPE/MnO_2_	DPV	0.1–10.0	27.4	-	[19]
^5^ PHLD- MCPE	CV	60–150	40.2	Pharmaceutical	[20]
^6^ FCNF/CNTPE	DPV	5.0–60.0	15.35	B-complex capsule	[21]
^7^ BiFE	SWAdSV	0.3–0.8	100	Oral solution, syrup, tablets	[22]
		1.0–9.0			
^8^ Fe_3_O_4_/rGO/GCE	DPV	0.030–1	89	Pharmaceutical, nutrition products	[23]
		1–100			
^9^ SnO_2_/RGO/GCE	SWV	0.1–150	34	Pharmaceutical, energy drink	[24]
^10^ ZnO/MnHCNF/GCE	DPV	0.2–3.0	0.0101	Pharmaceutical, milk powder	[25]
^11^ GO/Au/polyEAmVS/GCE	DPV	1–100	7.2	Non-alcoholic beverage, energy drink	[26]
^12^ Bi_2_WO_6_(PVP + NaOH)/GCE	DPV	0.03–457	3.65	Almond milk, soymilk	[27]
^13^ HGCE	SWV	0.01–0.07	5	Multivitamin tablets	[28]
		0.07–1.0			
^14^ DNA-PGE	DPV	1.86–133	956.5	Multivitamin tablets	[29]
^15^ PPGE	ASDPV	0.008–2.34	0.202	Multivitamin tablets	[29]
^16^ Sn/Cs/PGE	SWV	0.01–1.2	5.56	Tablets, milk powder	[30]
^17^ PGl/PGE	SWV	0.02–0.45	1.24	Pharmaceutical, foods	[31]
^18^ SPE	DPV	2.66–61.1	2390	Foods	[32]
^19^ sparked-BiSPEs	SWV	0.001–0.1	0.7	Pharmaceutical	[33]
^20^ N-CQD/SnO_2_/SPCE	DPV	0.05–306	8	Tablets, milk powder	[34]
^21^ AgNP-SPE	DPV	0.0019–0.1	0.56	Pharmaceutical, energy drink	[35]
^22^ OMC/GCE	CV	0.4–1.0	20	Vitamin tablets	[36]
^23^ GC/MWCNTs-Mn^III^salen	DPV	1.0–400	730	Injection, tablet	[37]
^24^ Pd-Cu@NSC/SPCE	DPV	0.004–0.1	0.0076	Tablets, milk powder	[38]
		0.02–9.0			
^25^ Ag/rGO/GCE	DPV	0.002–2.2	0.6	Pharmaceutical	[39]
^26^ USPtNPs-DES/MWCNT/GCE	SWV	0.02–1.2	1.8	Energy drink, biological fluids	[40]
^27^ PTN/GCE	DPV	0.01–65	3	Human plasma	[41]
^28^ PNNMGPE	LSV	5.0–65.0	782	Tablets	[42]
^29^ EP(VLN)MGPE	CV	2.0–40.0	286.9	Pharmaceutical	[43]
NiZY/CNHs-GCE	DPV	0.027–0.53	8.6	Dietary supplements	This work

^1^ Co^2+^-Y-zeolite-modified CPE; ^2^ manganese tetraphenylporphyrin-modified CPE; ^3^ manganese dioxide-modified CPE; ^4^ shungite paste electrode modified with MnO_2_ redox mediator; ^5^ polymerized-helianthin-dye-modified CPE; ^6^ functionalized carbon nanofiber and carbon nanotube composite paste electrode; ^7^ bismuth-film electrode; ^8^ Fe_3_O_4_ anchored reduced graphene oxide-modified GCE;^9^ tin oxide/reduced graphene oxide-modified GCE; ^10^ ZnO-manganese hexacyanoferrate-modified GCE; ^11^ graphene oxides/Au-nanoparticles/ionic liquid polymer of vinyl sulfonic acid sodium salt and methyl imidazolium-modified GCE; ^12^ bismuth tungstate (NaOH and poly(vinyl pyrrolidone)-modified GCE; ^13^ electrically heated graphite cylindrical electrode; ^14^ DNA-modified PGE; ^15^ pretreated PGE; ^16^ tin incorporated chitosan polymer matrix coated PGE; ^17^ polyglycine-coated PGE; ^18^ screen-printed electrode; ^19^ sparked-bismuth oxide SPE; ^20^ nitrogen-doped carbon quantum-dot-modified screen-printed carbon electrode; ^21^ Ag-nanoparticle-modified graphite SPE; ^22^ ordered mesoporous carbon-modified GCE; ^23^ multiwalled carbon nanotubes coupled with manganese salen-modified GCE; ^24^ palladium–copper nanoparticle-modified highly porous carbon electrode; ^25^ silver nanoparticles/reduced graphene oxide-modified GCE; ^26^ ultrasmall platinum nanoparticles synthesized in reline deep eutectic solvent/multi-walled carbon nanotube-modified GCE; ^27^ polythiophene-nanotube-modified GCE; ^28^ poly (niacin)-modified graphite paste electrode; ^29^ electrochemically polymerized valine-modified graphite paste electrode. SWAdSV—square-wave adsorption stripping voltammetry; ASDPV—differential pulse adsorptive stripping voltammetry; SWV—square-wave voltammetry.

**Table 5:** Results of VB_2_ and VB_9_ determination in dietary supplements.

Vitamin	Sample	Amount of VB_2_ [mg per Tablet]		RE [%]	RSD [%]
		Declared	$\mathrm{Found}\;\bar{x}$ ± *S*		
VB_2_	*Apteo Witamina B*_2_	3	2.90 ± 0.06	−3.3	2.1
	*Panawit Witamina B*_2_	10	9.38 ± 0.12	−6.2	1.3
VB_9_	*Olimp Labs Kwas foliowy*	0.4	0.411 ± 0.008	2.7	1.9
	*ActiFolin*	0.8	0.813 ± 0.003	1.6	0.4

$\bar{x}$—mean value; *s*—standard deviation; RSD% = *s*/$\bar{x}$ × 100%.

## Data Availability

The original contributions presented in this study are included in the article. Further inquiries can be directed to the corresponding author.
